# Facilitating manual wheelchair skills following lower limb amputation using a group process: A nested mixed methods pilot study

**DOI:** 10.1111/1440-1630.12759

**Published:** 2021-07-28

**Authors:** Kimberly Charlton, Carolyn Murray, Rose Boucaut, Angela Berndt

**Affiliations:** ^1^ Central Adelaide Local Health Network, Department of Health and Wellbeing South Australian Government, Hampstead Rehabilitation Centre Lightsview South Australia Australia; ^2^ Allied Health Science and Human Practice The University of Adelaide Adelaide South Australia Australia; ^3^ Allied Health and Human Performance, International Centre for Allied Health Evidence University of South Australia Adelaide South Australia Australia

**Keywords:** amputees, disability, motor skills, patient education, rehabilitation, wheelchairs

## Abstract

**Introduction:**

The manual wheelchair skills training programme is used to structure teaching manual wheelchair use for people following injury or disability. This pilot study aimed to explore the outcomes of introducing a group wheelchair skills training programme on skill performance, confidence and frequency of wheelchair use for people with lower limb amputation in a rehabilitation setting from the perspective of participants and group facilitators.

**Method:**

This pilot study used a two‐phase mixed methods nested design. Eleven people with lower limb amputations received a minimum of two 45‐min wheelchair skills sessions, using the Wheelchair Skills Training Program, delivered in a mix of group and one‐to‐one sessions. In phase one, wheelchair skill performance, confidence and frequency were measured using the Wheelchair Skills Test Questionnaire‐Version 5.0, goal achievement was measured through the Functional Independence Measure and Goal Attainment Scale. These measures were repeated in phase two. Nested within phase two was qualitative data collection. Interviews were conducted with eight participants and a focus group held with three programme facilitators, to gather their perceptions of the training process. Descriptive statistics were used to analyse and report quantitative data and thematic analysis was used to combine qualitative data from the two participant groups.

**Results:**

Post intervention, the mean Wheelchair Skills Test Questionnaire score increased in performance (42.3 ± 13.4), confidence (33.9 ± 20.7) and frequency (33.9 ± 27.3). Goal Attainment was achieved or exceeded by 91% of all participants. Four themes were developed from qualitative data including, “motivators driving learning,” “delivery methods, structure and profile of the Wheelchair Skills Training Program,” “managing risk and safety” and “confidence in wheelchair use.”

**Conclusions:**

The pilot study found that The Wheelchair Skills Training Program can improve wheelchair performance, confidence and frequency to support enhanced safety, independence and quality of life for people with lower limb amputations.

## 1 INTRODUCTION

Globally, an estimated 2,500 limb amputations are undertaken daily (Moxey et al., 2011). Most of these amputations are of the lower limb and related to peripheral vascular disease, neuropathy and soft tissue injury secondary to diabetes mellitus (Moxey et al., 2011). In the next 10 years, there is a proportionate rise expected in lower limb amputations amongst people with diabetes and an associated need for quality rehabilitation (Moxey et al., 2011).

The rehabilitation process varies depending on the cause of amputation (traumatic or vascular), the mental and physical capabilities of the individual and the potential of the amputated leg to be fitted with a prosthesis (Fiedler et al., 2014). Regardless of whether they will be a prosthetic user, most Australian people experiencing lower limb amputations are prescribed a manual wheelchair, in the anticipation that it will support independence with mobility (Fiedler et al., 2014; Gupta & Kumar, 2019). Simply providing a wheelchair does not equate to its safe and functional use. Ability to use a wheelchair is dependent on age, confidence, pain, strength and endurance, as well as environmental factors (Fiedler et al., 2014). Education about using a wheelchair is pivotal for independence and safety (Sakakibara et al., 2013).

Many wheelchair users and their carers receive insufficient wheelchair skills training (Best et al., 2015; Kirby et al., 2020). Concerningly, more than half of experienced community dwelling wheelchair users have reported one wheelchair related accident over a 3‐year period and 17% experienced two or more (Chen et al., 2011). Several factors contribute to inadequate wheelchair skills training, including limited time and resources of clinicians, limited length of stay, lack of training resources and low clinician confidence to demonstrate and teach (Kirby et al., 2020). Quantitative research suggests clinical confidence to teach wheelchair skills is improved with the use of a structured curriculum for wheelchair skills training and attendance at conferences, workshops, or in‐service training. However, clinician perspectives about these strategies are largely unknown (Giesbrecht, Wilson, et al., 2015b).

Competent use of a wheelchair supports independence in activities of daily living and return to work, reduces reliance on carers/families, avoids admission to long term care facilities, and positively impacts stress, social interaction and economic engagement (Best et al., 2015; Kirby et al., 2020). It is expected that implementation of a wheelchair skills training programme for wheelchair users may support development of their confidence and decrease tips and falls (Keeler et al., 2019; Kirby et al., 2020; MacPhee et al., 2004). One Canadian programme that has a growing body of evidence, is the wheelchair skills training program (WSTP). This includes the assessment and teaching of 32 different skills broken down into indoor, community and advanced (Kirby et al., 2018b).

According to two systematic reviews, WSTP has a clinically meaningful effect on wheelchair users' skill performance in the short term (Keeler et al., 2019; Tu et al., 2017). However, many of the included studies involved powered and experienced wheelchair users (Best et al., 2005; MacPhee et al., 2004; Routhier et al., 2012). Of the 13 studies outlined in the most recent systematic review (Keeler et al., 2019), there were only three studies including people with amputations amongst a mixed participant group. Two studies that included people with amputations demonstrated statistically significant improvements in skill from pre to post training (Best et al., 2005; MacPhee et al., 2004), and the other study suggested an increase in skill particularly with community wheelchair skills (Routhier et al., 2012). Outcomes were not analysed depending on diagnostic group, therefore more research exploring the effectiveness of the WSTP specifically for people with amputations is warranted. The studies that have investigated wheelchair skill training have focused primarily on wheelchair performance and confidence outcomes, but have not considered qualitative perspectives, including users' experiences of training and perceptions on individual or group training formats.

While the WSTP supports individual and group training formats, it is less clear whether one mechanism is more efficacious than the other. Two papers have reported on the facilitation of skills in a group setting, but these occurred with experienced community wheelchair users, largely with spinal cord injuries (Best et al., 2016; Worobey et al., 2016). Given the success of group programs to support people pre and post amputation (Marzen‐Groller & Bartman, 2005), clinician led group wheelchair skills training, that encourages support between group members was piloted in an inpatient rehabilitation setting in Australia.

The following research questions were explored through this pilot study:

1. What are the outcomes for people with lower limb amputation participating in group WSTP?
2. What are the perspectives of participants and facilitators about the process of the WSTP within the inpatient rehabilitation setting?

## 2 METHOD

### 2.1 Study design/ethics

**FIGURE 1:**
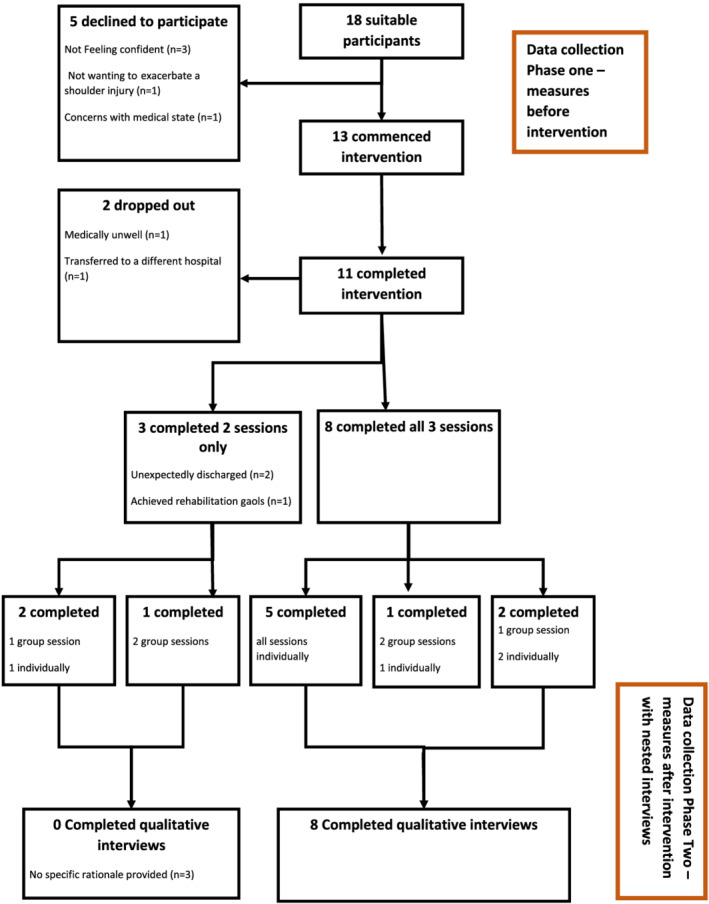
Participant recruitment strategy and overview of phases of data collection

A nested mixed method design (Creswell & Plano Clark, 2007) was used to collect pilot data across two phases (see Figure 1). This design is suitable for qualitatively evaluating the process of an intervention as well as quantitatively evaluating the outcomes. With this study design, the two types of data do not require integration during analysis and interpretation (Creswell & Plano Clark, 2007). A reporting guide for mixed methods was used to ensure the research occurred with rigour (Leech & Onwuegbuzie, 2010). The first phase involved the collection of quantitative baseline data for people with newly acquired lower limb amputations who were completing their rehabilitation at Hampstead Rehabilitation Centre. The second phase involved collection of quantitative post intervention data, and nested within this were individual semi‐structured interviews with a sub‐set of this sample. Within this phase the perspectives of group facilitators were also gathered through a focus group. The qualitative data were collected to augment the quantitative findings (Leech & Onwuegbuzie, 2010). Written consent was obtained from all WSTP participants and facilitators. The research was approved by Central Adelaide Local Health Network (CALHN) Human Research Ethics Committee and University of South Australia Human Research Ethics Committee Ref no. R20190129.

### 2.2 Participant recruitment and sampling

#### 2.2.1 Phase 1

Convenience sampling (Patton, 2002) was used to invite participation from people with newly acquired lower limb amputations who arrived on the rehabilitation ward between May 2019 and February 2020. To participate, they needed to be over 18 years old, have no aggressive behaviours, and use a self‐propelled manual wheelchair. Participants were excluded if they were acutely unwell, had a cognitive impairment that required direct 1:1 supervision or based on functional observation did not have sufficient upper limb coordination/strength to propel a manual wheelchair. Based on historical numbers of suitable people passing through the ward, a sample size of 24 was anticipated.

#### 2.2.2 Phase 2

After completion of the WSTP, a sub‐set of participants were purposively invited to an interview (Patton, 2002). To seek maximum variation in this sample, participants were selected that reflected diversity in confidence scores and skill performance (Patton, 2002). Using convenience sampling, all WSTP facilitators were invited to a focus group.

### 2.3 Intervention

The intervention was based on the WSTP‐ Version 5.0. (Kirby et al., 2018b). The WSTP is validated and has structured teaching of skills for wheelchair use. For this pilot, only indoor and community skills were taught thus excluding advanced skills and ascending and descending a low kerb. This decision was based on the highly variable admission length and the short time frames available for building up strength and endurance of wheelchair users, many of whom had chronic health comorbidities. This reduction left 21 of the 32 skills in the WSTP (Table 1).

**TABLE 1:** Manual wheelchair skills in WSTP (Kirby et al., 2018b)

No.	WSTP skill names included in this research	Skill level	No.	WSTP skill names excluded in this research	Skill level	
1	Rolls forward short distance and stops	Indoor	22	Ascends low curb	Community	
2	Rolls longer distance	Community	23	Descends low curb	Community	
3	Rolls backward short distance and stops	Indoor	24	Ascends high curb	Advanced	
4	Turns in place	Indoor	25	Descends high curb	Advanced	
5	Turns while moving forward	Indoor	26	Performs stationary wheelie	Advanced	
6	Turns while moving backward	Indoor	27	Turns in place in wheelie position	Advanced	
7	Manoeuvres sideways	Indoor	28	Rolls forward and backward in wheelie position	Advanced	
8	Picks objects from floor	Indoor	29	Descends high curb in wheelie position	Advanced	
9	Relieves weight from buttocks	Indoor	30	Descends steep incline in wheelie position and stops	Advanced	
10	Performs level transfers	Indoor	31	Ascends stairs	Advanced	
11	Folds and unfolds wheelchair	Community	32	Descends stairs	Advanced	
12	Performs ground transfers	Community
13	Gets through hinged door	Indoor
14	Ascends slight incline	Community
15	Descends slight incline and stops	Community
16	Ascends steep incline	Community
17	Descends steep incline and stops	Community
18	Rolls across side‐slope	Community
19	Rolls on soft surface	Community
20	Gets over obstacle	Community
21	Gets over gap	Community

### 2.4 Study procedure

The WSTP was delivered for 45 minutes for three consecutive weeks facilitated by one or two occupational therapists or allied health assistants. Circuits for skill acquisition around the grounds at the inpatient rehabilitation centre were determined prior to the pilot. Each facilitator shadowed the first author (KC) for two sessions before running sessions on their own. The first author has more than 5 years' experience in teaching wheelchair skills but all other facilitators had no prior experience. Facilitators were provided with written copies and links to the WSTP webpage. The day and the time of the WSTP remained flexible to accommodate participant availability. Over the three sessions, indoor skills were consolidated before progressing to community skills. In circumstances where only two sessions were completed, all indoor skills were covered, but sometimes there was insufficient time to fully address community skills. While the intent was to deliver WSTP in groups, this was not always possible with some one‐to‐one sessions occurring based on the availability of programme participants on the ward at the time of running the sessions. Participants who received individual training were able to have higher frequency of skill practice due to the facilitator only having one person to spot and all were encouraged to practice their skill acquisition in their own time if the occupational therapists agreed they were safe. The WSTP was completed using a manual wheelchair provided by the rehabilitation centre until participants sourced their own wheelchair.

### 2.5 Data collection tools

#### 2.5.1 Phase 1: Quantitative data collection

Following provision of consent, electronic health records were used to collect demographic data regarding: sex, age, weight, diagnosis, other comorbidities, living/social situation and time since amputation.

##### Wheelchair skills test—Questionnaire

The first 21 of the 32 skills outlined in the Wheelchair Skills Test—Questionnaire (WST‐Q) (Version 5.0) (Kirby et al., 2018a) were completed by participants pre and post intervention, with the treating occupational therapist providing support if required (Table 1). The remaining 11 skills were removed from the scoring. Pre‐testing was completed less than a week before the first WSTP session and post‐testing was completed less than a week post completion of the WSTP. The WST‐Q, is highly correlated with practical skill performance and is a self‐reported tool focusing on performance (the ability to do an activity in the everyday setting), confidence (to complete skills safely and consistently in own environment) and frequency (how often the skills were completed in their environment) (Kirby et al., 2018a). The WST‐Q provides a percentage score across three domains of capacity, confidence and performance. This tool was chosen over a practical test as it was less resource intensive and safe for participants prior to receiving their WTSP. The WST‐Q has been previously used in the early stages of rehabilitation to prioritise which wheelchair skills to address (Kirby et al., 2018a).

##### Goal attainment scale

The Goal Attainment Scale (GAS) was used to measure achievement of occupation specific wheelchair goals through quantifying the meaning of goal achievement in specific and measurable terms. GAS is being increasingly used as an outcome measure in rehabilitative research (Turner‐Stokes, 2009). Given the individual nature of goals, the GAS' psychometric qualities cannot validly be compared; however, it is suggested that the GAS has good content validity when used by experienced facilitators (Krasny‐Pacini et al., 2013). In this case, all goal setting was facilitated by the first author (KC). Participants were asked to focus on one major goal which was measured pre and post WSTP.

##### Functional Independence measure

Functional Independence Measure (FIM) scores relating to mobility were captured to track wheelchair mobility changes pre and post WSTP (Linacre et al., 1994). The FIM is widely used within rehabilitation settings and offers excellent content validity across 18 items on a seven‐point ordinal scale (Dodds et al., 1993).

#### 2.5.2 Phase 2: Qualitative data collection

##### People with amputations

Participants' perspectives about the WSTP were collected through semi structured interviews (Liamputtong, 2013) either face to face or by telephone within 2 weeks of completion of the WSTP. Due to limited availability of the independent research assistant, she only conducted one interview with the remaining seven interviews conducted by the first author (KC). A flexible topic guide was developed with seven open ended questions to gain participants overall perspectives of the WSTP, including areas of strength and improvement. The guide was developed by the second author (CM) and refined by the research team after piloting with four allied health professionals working with people with amputations (see supporting information).

##### Allied health professionals

All allied health professionals who had facilitated the WSTP participated in a focus group at the end of the 10 months of data collection. To reduce the introduction of bias, the first author (KC), was excluded from the focus group. The focus group was completed on the ward by the second author (CM), an experienced researcher external to the programme. A flexible focus group guide, encompassing seven questions was developed by the second author (CM) and refined by the research team. Facilitators were asked about their experience of the WSTP, and any perceived benefits, challenges and recommendations (see supporting information).

### 2.6 Data analysis

Descriptive statistics were used to analyse and report data from each domain of the WST‐Q (capacity, confidence and performance), FIM and GAS into frequency distributions. Mean calculations with standard deviation were used to report data distribution and range (DePoy & Gitlin, 2016). Comparative analysis was not completed due to insufficient sample size.

A qualitative descriptive approach was used to explore the perceptions of participants and facilitators (Stanley & Nayar, 2014). All interview and focus group data were digitally recorded and professionally transcribed. Data were analysed using reflexive thematic analysis (Braun & Clarke, 2012). Initially, the interview and the focus group transcripts were analysed separately but were then synthesised into themes that triangulated the data from both participant groups. Analysis involved independent line by line coding of all transcripts by the first author and duplicate coding of some transcripts by the second author. Following this, the research team met to discuss the coding before the first author clustered codes into categories. Seventeen categories were formed from initial coding of the interviews and a further 22 from the focus group. Further engagement with data, including returning to the original transcripts, enabled the inductive synthesis of findings into four themes (Liamputtong, 2013). Any differences in interpretation amongst the research team during the analysis process were resolved through discussion. A reflective journal detailing the first authors' preconceptions and assumptions was kept and reviewed during data analysis to minimise bias in interpretation of the data. An audit trail was recorded to track analytical decisions (Liamputtong, 2013). Pseudonyms were allocated for data reporting.

## 3 RESULTS

### 3.1 Participants

Eighteen people were invited to participate in the WSTP, five declined and two dropped out (see Figure 1). Of the 11 attendees of the WSTP, not all attended over three consecutive weeks or completed sessions in a group format. Eight of these attendees participated in qualitative interviews. Interviews lasted an average of 7 min (range 3.34–17.31).

The 11 participants had newly acquired amputations (see Table 2), 10 due to vascular related complications and one through traumatic events, and all were new wheelchair users. They commenced the WSTP an average of 19.6 ± 8.3 days post lower limb amputation. There were two females, nine males with an average age of 58.7 ± 15.9 (range 22–85 years).

**TABLE 2:** Results of WST‐Q

Pseudonym	Sex	Diagnosis traumatic/non‐traumatic	No. sessions attended	Mode of sessions	Method of interview	Percentage change in WST‐Q capacity	Percentage change in WST‐Q confidence	Percentage change in WST Q performance
Adam	M	Vascular‐below knee amputation	3	2 individual 1 group	Phone	26	2	4
Brad	M	Vascular‐ above knee amputation	3	2 individual 1 group	Face to face	19	14	18
Chris	M	Vascular‐ below knee amputation	3	3 individual sessions	Face to face	43	36	16
Dave	M	Vascular below knee amputation	3	1 individual 2 group	Face to face	44	47	33
Edward	M	Vascular‐ above knee amputation	3	3 individual	Face to face	69	69	71
Freddie	M	Traumatic amputation‐ below knee	3	3 individual	Face to face	36	36	90
Gerda	F	Vascular‐ above knee amputation	3	3 individual	Face to face	57	54	46
Henry	M	Vascular‐ above knee amputation	3	3 individual	Face to face	41	33	0
Ian	M	Vascular‐ below knee amputation	2	2 group	NA	43	32	29
Jack	M	Vascular‐ bilateral below knee amputation	2	1 individual 1 group	NA	46	30	42
Kate	F	Vascular‐ below knee amputation.	2	1 individual 1 group	NA	41	1	24

Two occupational therapists, two allied health assistants and one fourth year occupational therapy student provided the WSTP intervention. Between the WSTP and the focus group, the allied health assistants qualified as occupational therapists and attended the focus group along with one of the other occupational therapists who had less than 3 years' experience. Participants in the focus group had not been educated in wheelchair skills training beyond university and this programme.

### 3.2 Outcome measures

#### 3.2.1 Wheelchair skill test‐questionnaire

**FIGURE 2:**
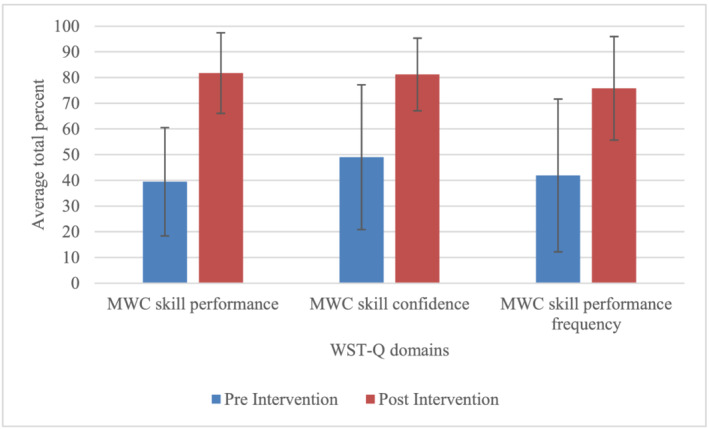
Average outcomes from the wheelchair skills test—Questionnaire (WST‐Q)—Performance, confidence, frequency

Participants reported perceived improvements in performance across indoor and community skills with a mean percentage increase at the completion of the WSTP of 42.3 ± 13.4. Similarly, confidence in completing the wheelchair skills was higher (mean percentage increase of 33.9 ± 20.7). After the programme, they more often completed wheelchair skills when they needed or wanted to (mean percentage increase of 33.9 ± 27.3). Table 2 details percentage of change for individuals and Figure 2 compares pre and post scores.

#### 3.2.2 Goal attainment scale

Due to the varied functional abilities and comorbidities experienced by participants, goals identified by participants varied considerably in task demand (i.e. complex community access to simple self‐propelling at home). Ten participants experienced improvement in their GAS, achieving or exceeding their identified goal, with an average GAS change of two points ±1. An example of goal achievement included one participant moving from being pushed by a therapist within his home environment to being able to independently navigate his home with increased time and effort. One participant did not achieve their goal but did not decline in GAS score.

#### 3.2.3 Functional Independence measure

Participants had an average mobility FIM change of 3.9 ± .5, with 82% (*n* = 9) recording a mobility FIM of six, meaning they could operate their wheelchair independently for a minimum of 50 m, turn around, manoeuvre the wheelchair to a table, bed and toilet, manoeuvre over rugs and over door sills and can negotiate a 3% graded ramp. The remaining two participants recorded a mobility FIM of five, indicating they could operate a wheelchair independently for short distances only, a minimum of 17 metres and there may be safety considerations or more time may be required to complete the task.

#### 3.2.4 Qualitative outcomes

The collective perspectives of programme participants (*n* = 8) and facilitators (*n* = 3) are described using four themes including: motivators driving learning; delivery methods; structure and profile of the WSTP; managing risk and safety and confidence in wheelchair use.

##### Theme 1: Motivators driving learning

There were conflicting opinions between participants and facilitators about the best way to learn wheelchair skills with participants not always being motivated.

“We had a few issues from patients who did not want to come” (Evie‐facilitator).

“Some people do not want to learn, want to sleep” (Chris‐participant)

According to facilitators, participants would engage sporadically and, on their terms, depending on who was in the group or their fatigue. Conflicting appointments and explanations of the programme influenced willingness to participate.

“you sort of pitch it that this is essential for your rehab and this is your main way of getting around and this is your mobility” (Maxine‐facilitator)

Communication and rapport building were essential in maintaining engagement in the programme as well as knowing the participants' capacity well enough to set small realistic challenges and provide skill specific positive reinforcement.

“I feel like rapport is a big one. … as I developed my relationship with him, I could see that he realised that all the tips I am giving him actually makes his life easier.” (Maxine‐facilitator)

Participants not knowing whether they were a candidate for a prosthesis meant they were uncertain about what they wanted to achieve, leading to facilitators being over‐involved in their goal setting.

“You're kind of like helping them create goals, so I guess it is probably a little bit skewed in regards to being really (emphasis) patient driven.” (Robin‐facilitator)

##### Theme 2: Delivery methods, structure and profile of the WSTP

Prior to the introduction of the WSTP, the training provided in wheelchair use was adhoc. Facilitators liked the structure of the WSTP and the manual to ensure that all key skills were covered.

“I found it really easy to just come in and facilitate the session with the handout” (Robin‐facilitator)

A large proportion of participants were ambivalent about the structure of sessions, including intensity and length. Freddie, Gerda and Edward found that the training grading and intensity were adequate and covered skills for a diverse age group, but Henry wanted more than one session a week to consolidate learning and Brad suggested sessions be longer to allow for the preservation of energy. One facilitator, Maxine, reflected that having three sessions provided flexibility to repeat and consolidate skills that participants found difficult. Maxine reported that session structure depended on the abilities of participants and their goals.

Participants additionally requested inclusion of wheelchair use during everyday tasks such as carrying objects when self‐propelling and suggested incorporating family members into training and provision of education around wheelchair maintenance.

“I think family members must know about wheelchair. I know family help to make lunch or something else, but they do not know how to help transfer from wheelchair to other chair.” (Chris‐participant)

Visual learning tools were highly valued as participants had difficulty following verbal instructions, particularly with some of the advanced skills that were difficult to explain.

“Seeing it done is way different to having someone say you have got to push here … it is a lot more instructional.” (Brad‐participant)

Facilitators developed a strategy of asking participants to visualise their wheelchair wheel as a clock to assist with hand positioning and propulsion and they suggested learning could be further facilitated through watching videos of skills being performed (i.e. iPad) either prior to or during WSTP.

All facilitators and four participants agreed that there was value in the WSTP occurring in a group setting as well as one‐to‐one due to the peer support that was offered.

“I love it when patients encourage other patients. It is more effective than when the therapists encourage them, just because they have that bond … in it together.” (Robin‐facilitator)

“you can bounce off each other and sort of share‐ do it with someone else and yeah help each other through.” (Freddie‐participant).

Brad, who had both individual and group sessions found it advantageous that the group format allowed for “*slowing down and having a bit of a rest and thinking about that next move.”* (Brad‐participant).

Facilitators reflected that it was rare to get concurrent eligible people with similar skill levels meaning they needed to be highly familiar with the process and participants. They suggested that low admissions during the time of the project exacerbated this issue. Some participants and facilitators wondered if one‐to one sessions were more advantageous because they could account for individual need.

“One‐to‐one is more focused, can be at the speed you are doing things” (Dave‐participant)

##### Theme 3: Managing risk and safety

Sound clinical reasoning skills and proactive planning and close positioning during training supported facilitators to manage risk during the WSTP.

“One time he actually did hit it (kerb) and almost went straight forward out of the wheelchair and … I was there to help prevent it from happening.” (Evie‐facilitator)

An example of clinical reasoning included weighing up the risk of making the chair more tippy by moving forward rear axle settings to decrease stress placed on shoulders to enhance propulsion technique.

Staff agreed that some level of risk helped participants learn more about their own ability to negotiate certain environments.

“Putting them in a situation where they are a little at risk, but with your support … then they have their own realisation that, no I should not be doing that” (Robin‐facilitator)

“Sometimes even though I wanted him to say he would not attempt something by himself, he would do it and so we'd do it together and then after he'd go OK actually that was really scary, I should not have done that.” (Maxine‐facilitator)

There were some conflicting opinions amongst the facilitators about confidence to deliver the WSTP. Robin reported feeling confident through drawing on knowledge learnt at university, observing colleagues and through referring to the adapted WSTP checklist. However, Evie felt that it was challenging to teach skills that she was only learning herself.

##### Theme 4: Confidence in wheelchair use

The WSTP was an opportunity to further assess the functional and cognitive performances of participants and link that back to decision making to facilitate discharge planning. Facilitators observed that participants who went through the WSTP were better equipped and more confident on discharge and this was affirmed by some participants.

“It's given me a bit of confidence to use it [wheelchair] wherever I am, you have got to know what you are doing up and down places and I do not want to flip sideways” (Freddie‐participant)

Two participants, Brad and Chris, spoke about feeling “scared” and anxious about using a wheelchair, particularly within a community setting, where it was perceived there was a higher potential risk of injury to themselves and others.

“I am scared …. Can I hurt someone maybe?” (Chris‐participant).

“Maybe I was too inexperienced with the wheelchair to be able to cope with the run offs and the footpaths …. I found it very scary.” (Brad‐participant)

Both facilitators and participants saw the value in graded skill acquisition to build confidence. Facilitators suggested training in more advanced skills such as negotiating shopping centres was warranted to further support confidence and reinforce basic skills as well.

“I guess in a more functional type setting that might be really handy. It goes hand in hand with OT [occupational therapy] does not it?” (Robin‐facilitator)

## 4 DISCUSSION

Findings of this research concur with earlier studies that suggest the WSTP builds wheelchair skill performance and confidence amongst wheelchair users (Keeler et al., 2019). Those involved in the WSTP emphasised its importance for the promotion of safe and independent wheelchair use and facilitators found the programme clinically relevant. Increased skill and confidence supported goal attainment, many of which related to occupational performance. The one participant whose GAS remained neutral still indicated increased confidence and skill according to their WST‐Q. This finding suggests that while the WSTP builds skills and confidence in wheelchair use, it does not always equate to the attainment of occupational performance goals. This is further supported in qualitative findings, with participants noting that the WSTP did not offer opportunity to practice wheelchair skills within everyday tasks.

Engagement in the WSTP was affected by challenges due to competing commitments and priorities, such as other rehabilitation or rest. It is unclear whether motivation played a role, or if the considerable impairment associated with a lower limb amputation and the process of coming to terms with the loss, may have interfered with their readiness to engage in rehabilitation and their willingness to engage in rich dialogue during interviews (Horgan & MacLachlan, 2004). There is a body of literature to suggest that goals within a rehabilitation setting are driven by a demand to empty hospital beds as quickly as possible creating tensions between different stakeholders (Siegert & Taylor, 2004). It is possible that within an inpatient rehabilitation environment, clinicians and participants are highly focused on specific wheelchair skills that support discharge, and do not consider more broadly how wheelchair skills fit into the performance of everyday occupations and occupational engagement. Alternatively, some people with amputations may still be unclear of their potential ability to use a prosthetic leg and may undervalue the need for wheelchair skill training within the acute rehabilitation setting or have difficulty envisaging themselves with the wheelchair outside of the hospital setting. This highlights the need for participants to be active in the goal setting process and for clinicians to encourage participants to think about their occupational performance once home thus working towards occupation‐based goals.

Implementation of this programme onto a busy rehabilitation ward was also challenging due to the high levels of staff rotations, limited staffing availability and competing time demands. Staff reported low facilitation confidence, indicating that the expectation of learning risk management processes and experiential skill development on the job is a flawed approach and that alternate mechanisms may be more efficacious.

A boot camp type approach that encompasses instruction, demonstration and hands on practice was identified as a successful method for learning the WSTP within Universities in North America (Giesbrecht, Wilson, et al., 2015b; Smith et al., 2020). However, this method involves taking significant time out of practice. Whilst not available when this pilot occurred, there is emerging research exploring flexible and comprehensive online learning for health professionals (Worobey et al., 2020). Additionally, tablet‐based wheelchair education sessions have been piloted to supplement education provided by health professionals and could be used for WSTP facilitation (Giesbrecht & Miller, 2019; Giesbrecht, Miller, et al., 2015a).

While it was recognised that group education enabled modelling and the provision of encouragement from others to support confidence, group education was challenging within the inpatient rehabilitation setting (Best et al., 2016). Alternate options, such as peer led sessions or including family or friends may be more practical solutions than provision of group education. This may be particularly advantageous given it has been recognised that carer training in wheelchair use has benefits (Kirby et al., 2020).

### 4.1 Limitations

As this was a pilot study with a low sample size, the intent was not to produce generalisable results. Participants in the WSTP were also receiving usual rehabilitation and experiencing natural recovery of function meaning improvements in WST‐Q and GAS cannot be attributed solely to the WSTP. Only 31% of WSTP sessions were completed in a group setting as anticipated, meaning participants received differing levels of attention and time spent practising wheelchair skills. The first author, who was involved in goal setting, the delivery of the programme and collection of data was not blinded which may have introduced some bias as well as affecting honesty from participants when interviewed. Additionally, there was no follow‐up once participants left rehabilitation to see carryover of skills learned in the home environment.

### 4.2 Implications for practice and future research

This research highlights the importance of a structured programme for teaching wheelchair skills for people with amputations and some of the associated systematic challenges. Facilitators require training in the delivery of wheelchair skills to support their confidence, but this training needs to be flexible to reduce the amount of time spent away from clinical practice. Raising the profile of WSTP as ‘usual practice’ and developing systematic protocols and processes related to the WSTP within inpatient rehabilitation settings is recommended and important for safety.

To support engagement with wheelchair skills training, it needs to be relevant to participants and this may be achieved through having a stronger occupational focus. The education and practising of more advanced wheelchair skills may be appropriate for completion within a community setting, where there may be stronger intrinsic motivation due to their environment (Siegert & Taylor, 2004). Exploration of the perspectives of people with amputations who are living within the community may provide insight into the optimal delivery and timing of the WSTP as well as give more information about the transference of wheelchair skills from the rehabilitation environment to home. Given the positive trends from this pilot study, larger scale research is recommended that includes a control group to enable between group comparison and clearer understanding of outcomes specific to the WSTP.

### KEY POINTS FOR OCCUPATIONAL THERAPY

- Structured education in wheelchair use builds skill capacity and confidence in people with amputations.
- An occupational focus may enhance wheelchair skills training.
- Occupational Therapists require flexible options for learning facilitation skills of WSTP

## CONFLICT OF INTEREST

The authors have no conflict of interest to declare.

## AUTHOR CONTRIBUTIONS

KC conceptualised and designed the research with support from other authors. KC and CM collected the data. Data analysis was led by KC with all authors contributing. All authors contributed to framing the write up of the manuscript and reviewed the final drafts. KC wrote the first full draft of the manuscript with CM contributing editing and revisions.

## Supporting information


**Data S1** Supporting Information

## Data Availability

Raw data were generated at Hampstead Rehabilitation Centre. Derived data supporting the findings of this study are available from the corresponding author [KC] on request.
