# Supplementary material for: Facilitating manual wheelchair skills following lower limb amputation using a group process: A nested mixed methods pilot study
Source: Aust Occup Ther J. 2021 Jul 28;68(6):490–503. doi: 10.1111/1440-1630.12759 (PMC9290744; doi:10.1111/1440-1630.12759)
Supplement: Supplementary file 1 — Data S1 Supporting Information [file AOT-68-490-s001.docx]

**Semi-structured Interview questions**

Tell me about your experience with the manual wheelchair skills training program on the ward at Hampstead?

What did you like about it? (prompts if needed - skills, staff, timing)

What do you think could be improved? (prompts if needed – skills, staff, timing)

How did you find the interactions with staff running the group?

What observations did you make of others in the group? Did they mention anything to you about the group?

Would you recommend the group to others?

How has the skills learned in the group helped you?

Any other comments/ thoughts to add?

**Focus group questions with staff**

Tell us about your experience with facilitating the manual wheelchair skills training program on the ward at Hampstead?

What did you think about the group process? What was good about the group? What could be improved?

What skills did you draw on to run the group? Did you feel that you had sufficient skills to run the group? Were you adequately prepared?

From a manual handling perspective, did facilitating the group pose any risks to you? Were you sore afterwards?

In what ways did you modify the training program to suit individual needs and goals?

What difference did participation in the group make to people with LL amputation? How did their involvement change your understanding of safety and risk for people with LL amputation?

Do you have any other comments/ thoughts to add?
